# Compositional profiling and bioefficacy studies of pulses-supplemented isocaloric designer biscuits for recently diagnosed diabetic individuals

**DOI:** 10.1016/j.fochx.2024.101305

**Published:** 2024-03-19

**Authors:** Iahtisham-Ul-Haq, Aqsa Akram, Iqra Yasmin, Hafiz Rizwan Sharif, Gulzar Ahmad Nayik, Seema Ramniwas, Shahida Anusha Siddiqui

**Affiliations:** a Kauser Abdulla Malik School of Life Sciences, Forman Christian College (A Chartered University), Lahore, Pakistan; b Department of Diet and Nutritional Sciences, Faculty of Allied Health Sciences, Imperial College of Business Studies (ICBS), Lahore, Pakistan; c Department of Human Nutrition and Dietetics, University of Chakwal, Chakwal, Pakistan; d Department of Food Science and Technology, Faculty of Science and Technology, University of Central Punjab, Lahore, Pakistan; e Department of Food Science & Technology, Government Degree College Shopian, 192303 Jammu and Kashmir, India; f University Centre for Research and Development, Chandigarh University, Gharuan, Mohali, Punjab, India; g Technical University of Munich, Campus Straubing for Biotechnology and Sustainability, Essigberg 3, 94315 Straubing, Germany; h German Institute of Food Technologies (DIL e.V.), Prof.-von-Klitzing Str. 7, 49610, Quakenbrück, Germany

**Keywords:** Chickpea, Designer biscuits, Type-2 diabetes mellitus, Glycemic index, Mung bean, Satiety index

## Abstract

This study addresses global concerns about diabetes mellitus by exploring a novel approach to manage hyperglycemia through pulses-supplemented designer biscuits. Control and designer biscuits were prepared with varying proportions of wheat flour and pulses (chickpea, mungbean). The pulses-supplemented biscuits exhibited increased protein content and reduced readily available carbohydrates. Selected designer biscuits, with 12.5 % incorporation of chickpea and mungbean pulse flour, demonstrated significantly lower glycemic index (69.17 ± 5.01) and higher satiety index (122.19 ± 8.85) compared to control biscuits. These showed 13 % less glycemic index and 9 % higher satiety index as compared to control biscuits. A four-week bio-efficacy trial involving diabetic subjects consuming these biscuits as a routine snack resulted in an 11.45 % decrease in fasting blood glucose and a 19.15 % reduction in random blood glucose levels. Insulin and HDL levels also significantly improved. The study concludes that these designer biscuits possess a hypoglycemic effect, offering a potential dietary intervention for managing diabetes.

## Highlights

• Low glycemic foods can be advised to manage diabetes mellitus.
• Pulses-supplemented designer biscuits were prepared.
• Chickpea and mungbean based biscuits had 13% lower GI and 9% higher satiety than controls.
• Diabetic subjects consuming these biscuits experienced an improved insulin and HDL levels.

## 1 Introduction

The trend for inclusion of plant-based diets to prevent, delay or improve diabetes mellitus in increasing nowadays instead of pharmaceutical medicine that may pose side effects. In this milieu, designer foods are important as they provide solutions to a health condition without negatively affecting the overall health of the individual. Designer foods are the foods tailored to prevent, delay or help in coping a targeted disease condition (Iahtisham-Ul-Haq, Butt, Shamshad, & Suleria, 2019).

For human nutrition, cereals are consumed as staple food and their incorporation into various foods holds great economic importance (Mellacheruvu, Talakayala, & Garladinne, 2019). The wheat (*Triticum aestivum L.*) is the most widely used cereal in developing countries. Rheological properties of dough that are related to baking quality are due to salient protein properties of the flour. Besides the beneficial nutrients content, the protein content in wheat is deficient in lysine, which is one of the essential amino acids (Pasha, Rashid, Anjum, Sultan, Qayyum, & Saeed, 2011). This amino acid deficiency can cause the dilemma of protein malnutrition specifically in developing countries, like Pakistan, where it is consumed as ample food (Giri et al., 2020, Hrušková and Švec, 2015). To overcome this dilemma, it is suggested that other cereals or grains should also be consumed that can fulfill the need of the deficient nutrients (V. Mishra et al., 2012, Mishra and Chandra, 2012).

Pulses belong to the grain *Leguminosae* family and are vital sources for dietary proteins consumed all over the world. Its consumption is predominant in communities that are unable to utilize animal proteins due to scarcity of meat, religious & cultural norms and poverty (Boye, Zare, & Pletch, 2010). Legumes are acknowledged as “meat for poor” being a cheap dietary source for proteins. Furthermore, these provide fibers, minerals, complex carbohydrates, and vitamins that are low in sodium and fats with no cholesterol (Sharif et al., 2018). They are also included among foods that possess low glycemic index. Among pulses, chickpea (*Cicer arientinum L.*) and mungbean (*Vigna Radiata L.*) are most important, as they not only possess significant dietary importance but also possess such properties that are therapeutically and medicinally recognized (Bravo-Núñez and Gómez, 2021, Kadam et al., 2012). Apart from providing source for essential amino acids and other bioactive compounds, many functional properties are also possessed by pulses that include water holding, fat binding and foaming, which boost up the prospective usage of pulses in wide range of food products (Boye, Zare, & Pletch, 2010). Furthermore, legumes provide low glycemic index products that are of vital importance for individuals suffering from various ailments such as diabetes, cardiovascular complications, and imbalanced lipid profiles (Di Cairano et al., 2020, Jenkins et al., 2012, Zhang et al., 2010). The legumes are considered as important dietary ingredients to prepare low glycemic food products and as these provide low glycemic index compared to cereal and pseudocereal flours (Di Cairano et al., 2020, Grdeń and Jakubczyk, 2023).

Epidemiological studies have predicted that 640 million adults aged between 20 and 79 years will be diabetic by the year 2040. Diabetes as well as its complications is posing a major threat to global health (Asmat, Abad, & Ismail, 2016). In 2015, a report given by International Diabetes Federation IDF showed a global estimation that 1 in 11 adults are diabetic. All forms of diabetes mellitus have been identified as one of the major causes that reduce the expectancy of life. Due to this reason diabetes mellitus is ranked 9th by the Global Burden of Disease Study 2013 (Zheng, Ley, & Hu, 2018). It has been estimated that 6.8 % of global mortality is caused by diabetes mellitus according to a 2010 report causing almost 4 million adults to die of this disease. The 2015 report estimated the raised level of deaths due to diabetes and its complications to be 5 million accounting for one death per six seconds. Globally, approximately 50 % of the cases of diabetes mellitus in adults remain undiagnosed, while frequently the disease’s onset occurs years before it is diagnosed (Zheng, Ley, & Hu, 2018). Diabetics who remain undiagnosed do not get proper treatment due to which they are at higher risk of getting the associated complications. The development of diabetes has been better understood by these epidemiological studies (Kharroubi and Darwish, 2015, Olfert and Wattick, 2018, Zheng et al., 2018).

Due to the demand of consumers for nutritious food products that are convenient as well, biscuits represent a fast-growing food segment. Longer shelf life, varied taste and relatively low cost are the factors that make biscuits a popular foodstuff that is consumed by a wide range of population (Irondi, Ajani, Aliyu, Olatoye, Abdulameed, & Ogbebor, 2021). Therefore, in order to improve the nutritive value of biscuits and to enhance their functionality, attempts are being made specially in modifying the nutrient content of the biscuits (Gani, Wani, Masoodi, & Hameed, 2012). One of the attempts involved preparation of different ranges of biscuits using a variety of flour blends. The evaluation of their nutritional quality showed promising role in helping to combat serious global issues like protein malnutrition (Okpala & Okoli, 2011).

Based on the mentioned facts, low glycemic foods can be advised to manage diabetes mellitus. However, the production of low glycemic food also lowers the net calories in the product due to which diabetic individuals may lose their weight. To cope with this challenge, isocaloric foods that can match the energy density but with lower glycemic index can be a promising strategy to curb both diabetes mellitus and unnecessary weight reduction. In this research, efforts were made to design isocaloric pulses-supplemented designer biscuits and their potential utilization for diabetic individuals. In this context, pulses-supplemented designer biscuits were prepared using chickpea and mungbean flours combined with wheat flour and their physicochemical, sensory, glycemic and satiety indices were studied. Moreover, the effect of these biscuits to manage diabetes mellitus was also studied.

## 2 Materials and methods

### 2.1 Production of designer biscuits

All the raw material required for the production of designer biscuits was procured from the local market at Lahore, Punjab, Pakistan. Both dry and wet ingredients were stored in air-tight jars and kept in cool and dry place until further use. The procured materials were prepared for product development (designer biscuits). The pulses were physically cleaned to remove any dust or miscellaneous particles. Further cleaning was mechanically done using a fine sieve. The cleaned pulses were then washed thoroughly under runny cold water and impurities were removed and then dried at 105 ± 2 °C for 2 h. After complete drying, they were sand-roasted and set to cool, after which they were ground to flour using electrical grinder (Panasonic MJ-W176P) and then sieved (30-mesh size sieve) separately to get uniform particle size. The prepared pulse flours were added to wheat flour as per treatment plan mentioned in Table 1 for making composite flours.

**Table 1:** Treatment plan for composite flours.

**Treatment**	**Wheat Flour (WF)** **(%)**	**Chickpea (CP) (%)**	**Mung bean (MB)** **(%)**
T**_0_**	100	__	__
T**_1_**	75	25	__
T**_2_**	50	50	__
T**_3_**	75	__	25
T**_4_**	50	__	50
T**_5_**	75	12.5	12.5
T**_6_**	50	25	25

**Fig. 1:**
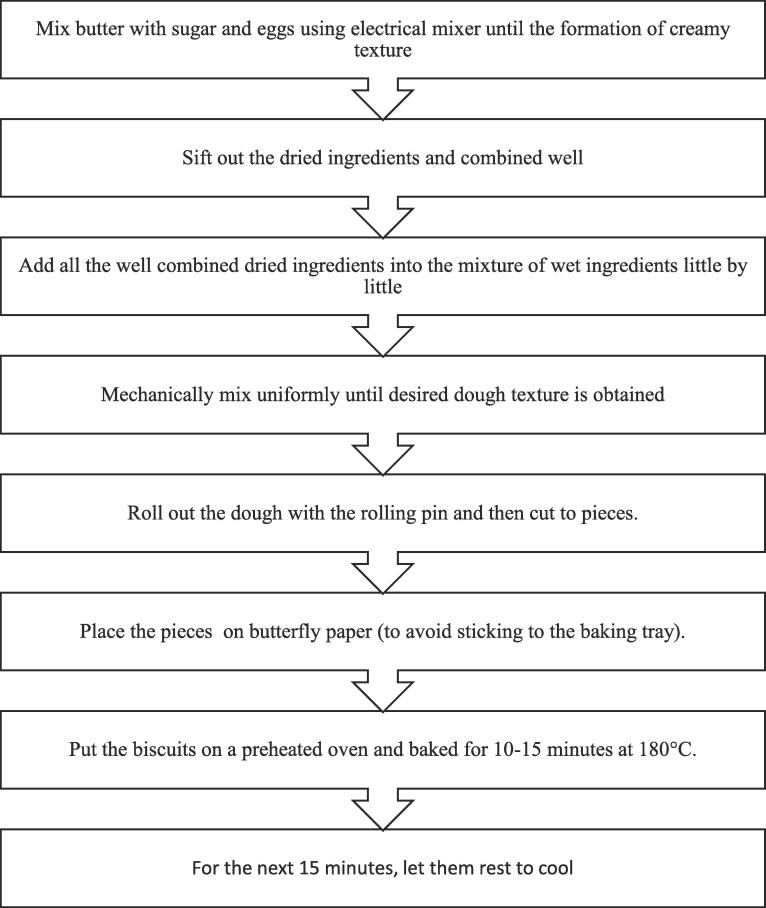
Preparation of designer biscuits.

According to the treatment plan, composite flours were used to prepare the designer biscuits by using standard recipe (Kaur, Sharma, Kumar, Panghal, Kaur, & Gat, 2017) detailed in Fig. 1.

### 2.2 Proximate composition of designer biscuits

The moisture (method No. 44–15.02), ash (method No. 08–01), protein (method No. 46–13), fat (method No. 30–25) and crude fiber contents of prepared designer biscuits were analyzed by following the methods as mentioned in AACC (2000). The nitrogen free extract (carbohydrate content) was calculated by subtraction of the sum of moisture, ash, protein, fat and crude fiber contents of the designer biscuits (Kaur, Sharma, Kumar, Panghal, Kaur, & Gat, 2017). By using factorial multiplication method, energy content was estimated (Tukassar et al., 2023). The expression followed is as under.

Energy (Kcal) = (4Kcal × Carbohydrates) + (4 Kcal × Proteins) + (9 Kcal × Fats)

### 2.3 Physical attributes of designer biscuits

The baked biscuits were assessed in terms of their weight on weighing balance and in terms of their diameter by measuring them with a calibrated scale. For the purpose, 6 biscuits from each treatment were selected at random and their weight and diameter were measured. The diameter was measured by arranging the biscuits edge to edge in a horizontal row. This way the average diameter was calculated. The height was measured by stacking 6 well-formed biscuits upon each other. The calculation of spread ratio was obtained by dividing diameter over height (Giwa & Abiodun, 2010).

### 2.4 Sensory evaluation of designer biscuits

Organoleptic properties of the biscuits including color, taste, aroma, texture, mouthfeel, and overall acceptability were evaluated by a panel comprising 10- members that were familiar with the product. Nine points hedonic scale was used with 9 being extremely liked and 1 as extremely disliked (Pasha, Rashid, Anjum, Sultan, Qayyum, & Saeed, 2011). The average score below 5 points was considered not acceptable for that sensory parameter.

### 2.5 Selection of designer biscuits for glycemic and satiety indices

Based on sensory acceptability, T_1_, T_3_ and T_5_ alongside T_0_ were selected for assessing their comparative glycemic and satiety indices.

### 2.6 Glycemic index of designer biscuits

Determination of glycemic index was done using the scientific approach of assessing glycemic response since the value of glycemic index is expressed as percentage of glycemic response. For this purpose, five groups were made, comprising of 10 healthy human subjects with even distribution of males and females in each group. The evaluation of glycemic index was done after 12 h of fasting period. Amongst the groups, the 1st group was provided with 50 g glucose equivalent from Glaxose-D® mixed in 250 mL water, the other groups were provided with respective biscuits i.e., T_0_, T_1_, T_3_ and T_5_, separately delivering the same amount of glucose per serving. The blood glucose concentrations of the subjects were noted pre- & post-product consumption after 30 min intervals. The value of glycemic index is calculated using the following equation (Alongi et al., 2019, Brouns et al., 2005, Vega-López et al., 2018).

$$GlycemicIndex\left(GI\right)=\frac{{\mathrm{AUC}}_{b}}{{\mathrm{AUC}}_{r}}\times 100$$

Where; AUC_b_ = area under the curve of biscuits.

AUC_r_ = area under the curve of reference glucose.

### 2.7 Satiety index of designer biscuits

For the assessment of satiety index, visual analogue scale (VAS) was used to record the response against satiety level for each treatment. For this purpose, five groups were made, comprising of 10 healthy human subjects with even distribution of males and females in each group. Amongst the groups, the reference group was provided with white bread (400 kcal), while other groups were provided with respective biscuits i.e., T_0_, T_1_, T_3_ and T_5_, separately delivering the same amount of energy as of white bread per serving. The respondents rated their satiety degree on a graph comprising of a numerical VAS consisting of 0–100 mm length for describing level of satiety was provided to each participant along with the blindly labeled samples. The scale estimates were no fullness (0 mm), slight fullness (0–20 mm), mild fullness (20–40 mm), moderate fullness (40–60 mm), extreme fullness (60–80 mm) and unbearable fullness (80–100 mm) Subjects were asked to rate their satiety score on that scale after the consumption of reference food and designer biscuits at time interval of 0 to 120 min with scale provided to them after every thirty minutes. Following the ingestion of test food, area under the curve (AUC) of satiety ratings was measured, which was then compared with the AUC of satiety ratings obtained after the consumption of a reference food (Drapeau et al., 2007, Tremblay and Bellisle, 2015). The expression is given below.

$$SatietyIndex\left(SI\right)=\frac{{\mathrm{AUC}}_{b}}{{\mathrm{AUC}}_{r}}\times 100$$

Where; AUC_b_ = area under the curve of biscuits.

AUC_r_ = area under the curve of reference food.

### 2.8 Study design

The study was conducted according to the ethical principles of the Declaration of Helsinki, and all participants gave written informed consent. This clinical trial was registered with the Ethical Review Board, Imperial College of Business Studies, Lahore, Punjab, Pakistan (Registration No. PHND02163006, Dated 07–11-2018). The hypoglycemic effect of selected designer biscuit was assessed using experimental trials on diabetic human subjects after consent from participants and ethical approval from Ethical Review Board, Imperial College of Business Studies, Lahore, Punjab, Pakistan. For the purpose, two parallel studies (normal and diabetic) were conducted each comprising two groups i.e., N_0_ & N_1_ as control & treated group in normal participants-based study, and D_0_ & D_1_ as diabetic control & diabetic treated group in diabetic participants-based study (Table 2). Each group consisted of ten subjects. The trial period was four weeks. Before the conduct of experiments, written informed consent was obtained from the subjects and their verbal consent was taken every time before drawing their blood samples for biochemical analysis. A diet plan of 1600 Kcal was prescribed to the diabetic subjects to be followed throughout study in addition to the designer biscuits (400 Kcal). The inclusion criteria for the participants consisted of normal individuals who were healthy and diabetic individuals who were recently diagnosed with diabetes mellitus according to the criteria of ADA (2015) were included in the study. For both normal and diabetic individuals, it was necessary that they were not taking any kind of medication so that there may not be any interaction of the dietary regime with the medicine and ensure the safety of the individuals. The age group of these individuals ranged between 20 and 40 years. Individuals with renal and other metabolic dysfunctions alongside diabetic individuals having any allied complication were excluded.

**Table 2:** Bio-efficacy plan.

**Studies**	**Treatment Groups**	**Description**
Normal subjects (Study 1)	N_0_	Normal Control
	N_1_	Normal subjects consuming designer biscuit
Diabetic subjects (Study 2)	D_0_	Diabetic Control
	D_1_	Diabetic subjects consuming designer biscuit

### 2.9 Serum glucose and insulin

The serum glucose levels of the subjects were evaluated using the method described by Kim, Paik, Kim, Park, Lee, Jang, et al. (2011), and insulin levels of the subjects were evaluated following the method by Ahn, Choi, Kim, and Ha (2011). In both normal and diabetic individuals, blood samples were collected in fasting and postprandial states. The analyses were repeated on a fortnightly basis.

### 2.10 Lipid profile

Blood lipids were measured from the blood samples collected from the subjects. Centrifugation of blood samples at 3000 rpm for 8 min was carried out to separate the serum. In the serum, the concentration of total cholesterol and triglycerides were measured using commercially available Fluitest TG (Triglyceride GPO-PAP) and Fluitest Chol (Cholesterin CHOD-PAP) kits (Biocon, Vőhl-Marienhagen, Germany). The concentration in supernatants of HDL-C (high density lipoprotein cholesterol) and LDL-C (low density lipoprotein cholesterol) were measured enzymatically after precipitating chylomicrons of the sera with dextran magnesium sulfate (Kim, et al., 2011) using Ecoline kits (Merck, Germany).

### 2.11 Physiological safety tests

The sera of the subjects were analyzed for bilirubin, Aspartate aminotransferase (AST), alanine aminotransferase (ALT) and Alkaline Phosphatase (ALP) levels (Martin and Friedman, 2017, Ni et al., 2012) to assess the liver health. Urea (Bamanikar, Bamanikar, & Arora, 2016) and creatinine (Dabla, 2010) were analyzed using automatic chemistry analyzer to assess the renal health of the individuals.

### 2.12 Statistical analysis

All the data regarding each parameter were assessed using Statistix 8.1. Analysis of variance (ANOVA) under completely randomized design was used to check the level of significance (P ≤ 0.05). Least significant difference test was performed for post hoc comparison of means. The correlation analysis was conducted using Microsoft Excel (Office 365).

## 3 Results

### 3.1 Compositional profile of designer biscuits

The nutritional composition of all designer biscuits is presented in Table 3. Protein and fiber contents of designer biscuits were comparatively higher, whereas total carbohydrates were comparatively lower compared to control (T_0_). High content of proteins and fibers in pulse flour contributes greatly to water holding capacity in composite flours, due to which there was slight decrease observed with moisture content in prepared designer biscuits. With respect to ash content, significant difference (p < 0.01) was observed, ranging from 2.15 ± 0.04 to 2.74 ± 0.05 g/100 g. The highest value among this range was observed with T_4_ and T_6_ having 50 % incorporation of mungbean in wheat flour and 25 % incorporation of each mungbean and chickpea flour, respectively. For protein and fiber contents of all the treatments particularly the treatment T_2_, T_4_ and T_6_ were 10.33 ± 0.21, 10.66 ± 0.21 and 10.50 ± 0.21 g/100 g, respectively as compared to control (7.60 ± 0.15 g/100 g). Also, the fiber content of T_2_, T_4_ and T_6_ was 3.19 ± 0.06, 4.43 ± 0.09 and 3.81 ± 0.08 g/100 g, respectively as compared to T_0_ (1.40 ± 0.03 g/100 g).

**Table 3:** Nutritional composition of pulses-supplemented designer biscuits.

**Treatments**	**Moisture** **(%)**	**Ash** **(%)**	**Protein** **(%)**	**Fat** **(%)**	**Total Carbohydrates** **(%)**	**Available Carbohydrates** **(%)**	**Fiber** **(%)**	**Energy** **(Kcal/100 g)**
T_0_	9.15 ± 0.18^a^	2.15 ± 0.04^c^	7.60 ± 0.15^c^	20.50 ± 0.41^b^	53.82 ± 1.08^a^	52.47 ± 1.05^a^	1.36 ± 0.03 ^g^	427.39 ± 8.55^a^
T_1_	9.08 ± 0.18^a^	2.42 ± 0.05^b^	8.97 ± 0.18^b^	21.15 ± 0.42^ab^	51.73 ± 1.03^ab^	49.46 ± 0.99^b^	2.27 ± 0.05^f^	428.37 ± 8.57^a^
T_2_	9.01 ± 0.18^a^	2.68 ± 0.05^a^	10.33 ± 0.21^a^	21.80 ± 0.44^a^	49.64 ± 0.99^b^	46.45 ± 0.93^c^	3.19 ± 0.06^c^	429.35 ± 8.59^a^
T_3_	9.03 ± 0.18^a^	2.47 ± 0.05^b^	9.13 ± 0.18^b^	20.52 ± 0.41^b^	52.28 ± 1.05^ab^	49.38 ± 0.99^b^	2.89 ± 0.06^d^	424.27 ± 8.49^a^
T_4_	8.91 ± 0.18^a^	2.79 ± 0.06^a^	10.66 ± 0.21^a^	20.54 ± 0.41^b^	50.73 ± 1.01^b^	46.30 ± 0.93^c^	4.43 ± 0.09^a^	421.14 ± 8.42^a^
T_5_	9.05 ± 0.18^a^	2.44 ± 0.05^b^	9.05 ± 0.18^b^	20.84 ± 0.42^ab^	52.00 ± 1.04^ab^	49.42 ± 0.99^b^	2.58 ± 0.05^e^	426.32 ± 8.53^a^
T_6_	8.96 ± 0.18^a^	2.74 ± 0.05^a^	10.5 ± 0.21^a^	21.17 ± 0.42^ab^	50.19 ± 1.00^b^	46.38 ± 0.93^c^	3.81 ± 0.08^b^	425.24 ± 8.50^a^

Values sharing similar alphabetical letters in each parameter are statistically alike (P > 0.05).

T_0_ = Control (WF:CP:MB; %100:0:0).

T_1_ = WF:CP:MB %(75:25:0).

T_2_ = WF:CP:MB %(50:50:0).

T_3_ = WF:CP:MB %(75:0:25).

T_4_ = WF:CP:MB %(50:0:50).

T_5_ = WF:CP:MB %(75:12.5:12.5).

T_6_ = WF:CP:MB %(50:25:25).

### 3.2 Physical analysis of pulses-supplemented designer biscuits

The statistical results indicated that weight, height and spread factor differed significantly by difference in concentration of pulses in composite flours while the diameter remained non-significant. Increased weight was observed with the treatments T_2_, T_3_, T_4_ and T_6_ with average values of 14.93 ± 0.30, 13.21 ± 0.26, 13.45 ± 0.27 and 14.01 ± 0.28 g/100 g, respectively as compared to that of control treatment T_0_ that had average value for weight as 11.93 ± 0.24 g. Control treatment T_0_ showed height of 0.51 ± 0.01 mm, while a decreased height was observed in the treatment T_2,_ T_4_ and T_6_ that was 0.41 ± 0.01, 0.38 ± 0.01 and 0.41 ± 0.01 mm, respectively. Fats added in preparation of biscuits act as shortening agents that can double the biscuits in terms of height. As compared to the control treatment, height in other treatments of designer biscuits decreased. Highest value of spread factor was observed in treatments T_2,_ T_4_ and T_6_ as 11.53 ± 0.23, 13.00 ± 0.26 and 12.13 ± 0.24, respectively, as compared to the control treatment T_0_ that resulted as 09.50 ± 0.19 (Table 4).

**Table 4:** Physical quality attributes of pulses-supplemented designer biscuits.

**Treatment**	**Weight (g)**	**Diameter (cm)**	**Height (cm)**	**Spread Factor**
T_0_	11.93 ± 0.24^e^	4.83 ± 0.1^a^	0.51 ± 0.01^a^	09.50 ± 0.19^d^
T_1_	12.99 ± 0.26 ^cd^	4.93 ± 0.1^a^	0.48 ± 0.01^b^	10.21 ± 0.20^c^
T_2_	14.93 ± 0.30^a^	4.98 ± 0.1^a^	0.43 ± 0.01^c^	11.53 ± 0.23^b^
T_3_	13.21 ± 0.26^c^	4.88 ± 0.1^a^	0.46 ± 0.01^b^	10.67 ± 0.21^c^
T_4_	13.45 ± 0.27^bc^	4.95 ± 0.1^a^	0.38 ± 0.01^d^	13.00 ± 0.26^a^
T_5_	12.42 ± 0.25^de^	4.90 ± 0.1^a^	0.46 ± 0.01^b^	10.72 ± 0.21^c^
T_6_	14.01 ± 0.28^b^	4.93 ± 0.1^a^	0.41 ± 0.01^c^	12.13 ± 0.24^b^

Values sharing similar alphabetical letters in each parameter are statistically alike (P > 0.05).

T_0_ = Control (WF:CP:MB; %100:0:0).

T_1_ = WF:CP:MB %(75:25:0).

T_2_ = WF:CP:MB %(50:50:0).

T_3_ = WF:CP:MB %(75:0:25).

T_4_ = WF:CP:MB %(50:0:50).

T_5_ = WF:CP:MB %(75:12.5:12.5).

T_6_ = WF:CP:MB %(50:25:25).

### 3.3 Sensory scores for pulses-supplemented designer biscuits

**Fig. 2:**
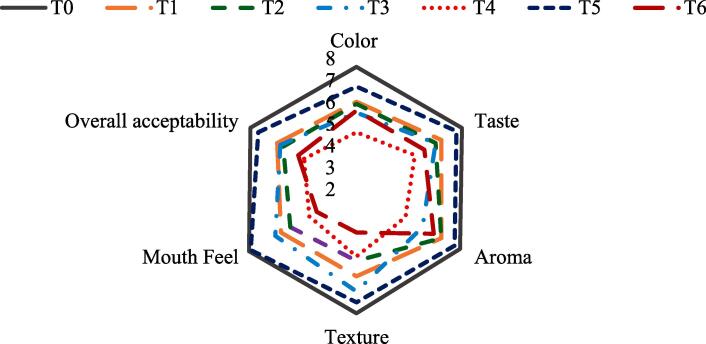
Mean scores for sensory quality attributes of designer biscuits.

Fig. 2 summarizes the obtained results with respect to the sensorial quality of designer biscuits. The data depicted that composite flour incorporation shows marked variations in color and textural profile of prepared designer biscuits. The color of designer biscuits became darker with the increase in concentrations of chickpea and mungbean flour that could be attributed to Maillard reaction products. According to panelists scoring, treatment T_1_, T_2_ and T_5_ scored the best values of 6.0 ± 0.88, 5.9 ± 0.88 and 6.7 ± 1.09, respectively. While the rest of the treatments acquired the lowest scores as compared to control treatment T_0_ that acquired a mean score of 7.6 ± 0.52. Mean score of color declined because of increased substitution level.

Preceded by control T_0_ with mean score of 7.6 ± 0.52, comparatively higher mean scores were acquired for taste by the treatments T_1_, T_2_, T_3_ and T_5_ as 6.5 ± 0.94, 6.2 ± 0.42, 6.2 ± 0.79 and 7.3 ± 1.05, respectively. The remaining treatments T_4_ and T_6_ acquired lower scores as compared to normal. It is noticed that incorporation of pulse flour was inversely related to the taste scores as treatments T_1_, T_3_ and T_5_ contain 25 % of flour replacement with pulse flour and scored more, while the other treatments were higher in pulse flour i.e.*,* 50 % and scored less.

Among the means of aroma scores, other than the control treatment T_0_ with mean scores of 7.5 ± 0.53, treatments T_1_, T_2_, T_5_ and T_6_ had maximum scores as 6.5 ± 0.52, 6.5 ± 0.53, 7.2 ± 0.99 and 6.1 ± 0.88, respectively. On the other hand, T_3_ and T_4_ obtained the minimum scores as 5.5 ± 0.53 and 4.6 ± 0.52. The quality of raw materials used in biscuits processing influences the aroma characteristic. The presence of vanilla essence significantly influenced the aroma characteristic of prepared designer biscuits. Textural characteristics of designer biscuits change significantly by variation in the inclusion ratio of different pulses over the control sample. Overall acceptability of designer biscuits was justified with the textural profile. Up to 25 % incorporation of composite flour to designer biscuits showed slight improvement in crispiness of biscuits, hence better scores were secured as compared to the designer biscuits containing 50 % of incorporated composite flour.

### 3.4 Glycemic and satiety profile of designer biscuits

Based on overall acceptability of designer biscuit’s, this can be concluded that incorporation of 12.5 % of chickpea and mung bean flours in composite flour preparation is superior to control sample and in all other treatments with respect to sensorial quality characteristics such incorporation could be considered optimum. Other than the control treatment T_0_, the treatments T_1_, T_3_ and T_5_ secured best mean scores for the sensory attributes of texture, mouthfeel and overall acceptability. Thus, these treatments were selected for further evaluation for glycemic and satiety indices.

**Fig. 3:**
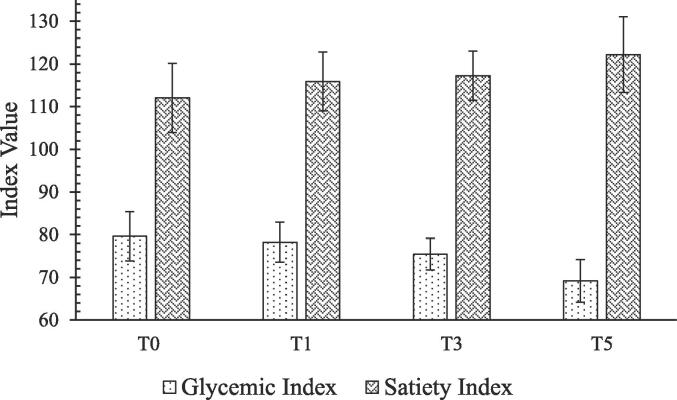
Glycemic index and satiety index of selected designer biscuits [T_0_ = Control (WF:CP:MB; 100:0:0); T_1_ = WF:CP:MB %(75:25:0); T_3_ = WF:CP:MB %(75:0:25); T_5_ = WF:CP:MB %(75:12.5:12.5)].

**Fig. 4:**
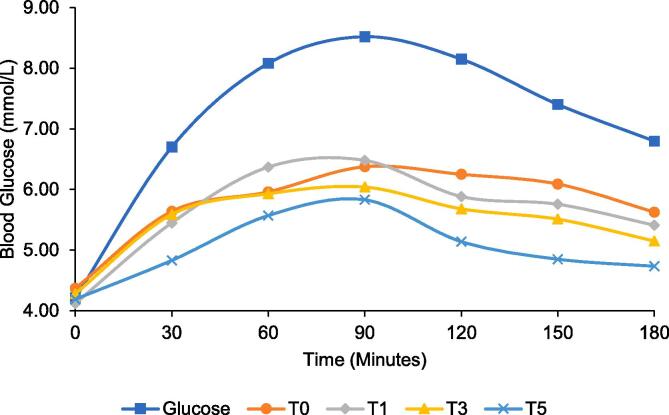
Average glycemic response of designer biscuits.

Fig. 3 summarizes the mean values of glycemic index for best-selected treatments of designer biscuits. The glycemic index of control biscuits (T_0_) was found to be 79.61 ± 5.77, while glycemic indices of T_1_, T_3_ and T_5_ were 78.24 ± 4.68, 75.45 ± 3.73 and 69.17 ± 5.01, respectively. Average glycemic response of designer biscuits is shown in Fig. 4. Mean glycemic index of treatments of designer biscuits was extremely lower as compared to the control.

**Fig. 5:**
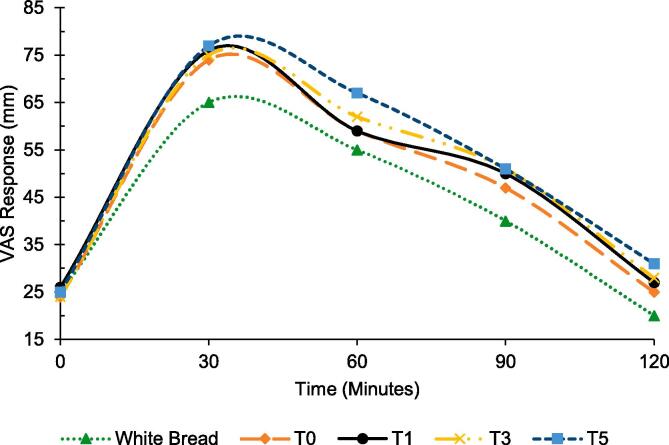
Average satiety response of designer biscuits.

The satiety index of T_0_ was 112.05 ± 8.12, while that of T_1_, T_3_ and T_5_ were 115.89 ± 6.93, 117.26 ± 5.79 and 122.19 ± 8.85, respectively (Fig. 3). Average satiety response of designer biscuits is shown in Fig. 5. The correlation analysis of the glycemic and satiety indices indicated a very strong negative correlation (-0.965) indicating that lower glycemic index yields in higher satiety index. Based on glycemic and satiety indices of designer biscuits, T_5_ prepared from 12.5 % of each pulse flour i.e., chickpea and mung bean and 75 % of wheat flour with low glycemic index as 69.17 ± 5.01 and high satiety index 122.19 ± 8.85 was selected for bio-efficacy trials in human subjects.

### 3.5 In vivo evaluation of designer biscuits

#### 3.5.1 Glycemic profile

##### 3.5.1.1 Fasting serum glucose

Significant variations were indicated by statistical analysis of fasting serum glucose because of the treatment and time intervals along with their interaction in study 2, while in study 1, significant variations were noted for fasting serum glucose because of the time intervals. In normal subjects (study 1), as the effect of treatment remained statistically alike, the pooled mean values of fasting glucose 88.70 ± 5.38 mg/dL in control subjects and 85.96 ± 6.42 mg/dL in treated subjects (Table 5). The mean values for effect of intervals on fasting serum glucose level of normal and diabetic subjects was observed as 90.57 ± 5.49 mg/dL at 0 day which reduced to 85.06 ± 5.92 mg/dL after 28 days (Table 6). Provision of designer biscuits showed reduction of fasting serum glucose level over twenty-eight days. Likewise, within study 2, diabetic control subjects represented 128.06 ± 5.18 mg/dL of fasting glucose that was significantly higher than diabetic subjects treated with designer biscuits representing 124.79 ± 7.73 mg/dL of fasting glucose. Diabetic control subjects in study 2 and those consuming designer biscuits showed elevated blood glucose levels for fasting as 131.24 ± 4.90 and 132.17 ± 4.93 mg/dL, respectively. During the study at initial day, the serum glucose of control subjects decreased from 131.24 ± 4.90 mg/dL to 125.82 ± 4.69 mg/dL after 28 days (Table 7). With designer biscuit incorporation, there was a reduction from 132.17 ± 4.93 to 117.04 ± 4.37 mg/dL over the 28 days period (Table 7). It may be concluded from the study that the designer biscuit treatment resulted in the highest reduction of fasting glucose by 4.13 % in diabetic subjects (study 2), while reduction of 9.8 % was observed in the same study on designer biscuit provision in normal subjects.

**Table 5:** Pooled means for effect of treatments on glycemic and lipid profile of normal and diabetic subjects.

**Studies**	**Treatments**	**Fasting Glucose** **(mg/dL)**	**Random Glucose** **(mg/dL)**	**Insulin** **(IU/L)**	**Low density lipoproteins** **(mg/dL)**	**High density lipoproteins** **(mg/dL)**	**Triglycerides** **(mg/dL)**	**Total cholesterol** **(mg/dL)**
Normal subjects (Study I)	N_0_	88.70 ± 5.38^a^	112.82 ± 6.80^a^	15.72 ± 0.95^a^	94.47 ± 5.71^a^	38.45 ± 2.34^a^	120.89 ± 7.24^a^	169.73 ± 10.42^a^
	N_1_	85.96 ± 6.42^a^	110.25 ± 7.87^a^	15.26 ± 1.23^a^	88.45 ± 7.17^b^	39.49 ± 2.38^a^	119.03 ± 7.46^a^	157.94 ± 14.67^b^
Diabetic subjects (Study II)	D_0_	128.06 ± 5.18^a^	210.42 ± 8.38^a^	14.71 ± 0.64^b^	123.78 ± 4.59^a^	33.11 ± 1.42^b^	144.33 ± 5.27^a^	215.29 ± 10.3^a^
	D_1_	124.79 ± 7.73^b^	198.6 ± 18.87^b^	15.82 ± 1.00^a^	115.42 ± 9.02^b^	34.55 ± 1.75^a^	142.34 ± 5.80^a^	209.18 ± 14.05^b^

Values with similar alphabetical letters in a column are statistically alike (P > 0.05) only within respective study.

N_0_ = Normal Control; N_1_ = Normal subjects consuming designer biscuit; D_0_ = Diabetic Control; D_1_ = Diabetic subjects consuming designer biscuit.

**Table 6:** Pooled means for effect of intervals on glycemic and lipid profile of normal and diabetic subjects.

**Studies**	**Intervals** **(days)**	**Fasting Glucose** **(mg/dL)**	**Random Glucose** **(mg/dL)**	**Insulin** **(IU/L)**	**Low density lipoproteins** **(mg/dL)**	**High density lipoproteins** **(mg/dL)**	**Triglycerides** **(mg/dL)**	**Total cholesterol** **(mg/dL)**
Normal subjects (Study I)	0	90.57 ± 5.49^a^	115.13 ± 7.01^a^	15.05 ± 1.27^b^	94.83 ± 5.72^a^	39.11 ± 2.36^a^	121.79 ± 7.34^a^	172.81 ± 10.41^a^
	14	86.35 ± 5.52^b^	110.19 ± 6.96^b^	15.55 ± 0.95^ab^	91.48 ± 6.60^ab^	38.83 ± 2.40^a^	119.59 ± 7.32^a^	161.96 ± 12.50^b^
	28	85.06 ± 5.92^b^	109.28 ± 7.19^b^	15.86 ± 1.00^a^	88.06 ± 7.52^b^	38.96 ± 2.55^a^	118.50 ± 7.36^a^	156.74 ± 14.07^b^
Diabetic subjects (Study II)	0	131.71 ± 4.81^a^	216.74 ± 8.02^a^	14.48 ± 0.58^b^	124.73 ± 4.57^a^	33.62 ± 1.30^a^	145.57 ± 5.30^a^	223.74 ± 8.14^a^
	14	126.13 ± 4.69^b^	205.10 ± 8.50^b^	15.52 ± 0.95^a^	120.08 ± 5.38^b^	33.71 ± 1.37^a^	143.02 ± 5.49^a^	211.54 ± 8.87^b^
	28	121.43 ± 6.31^c^	191.68 ± 17.12^c^	15.80 ± 0.93^a^	113.98 ± 10.03^c^	34.16 ± 2.39^a^	141.42 ± 5.43^a^	201.42 ± 9.19^c^

Values with similar alphabetical letters in a column are statistically alike (P > 0.05) only within respective study.

**Table 7:** Glycemic and lipidemic response of pulses-supplemented designer biscuits in normal and diabetic individuals.

**Studies**	**Groups**	**Intervals** **(days)**	**Fasting Glucose** **(mg/dL)**	**Random Glucose** **(mg/dL)**	**Insulin** **(IU/L)**	**Low density lipoproteins** **(mg/dL)**	**High density lipoproteins** **(mg/dL)**	**Triglycerides** **(mg/dL)**	**Total Cholesterol**
Normal subjects (Study 1)	N_0_	0	90.01 ± 5.57^a^	114.12 ± 7.06^ab^	15.91 ± 0.99^a^	95.14 ± 5.89^a^	39.01 ± 2.42^a^	121.55 ± 7.52^a^	172.74 ± 10.69^a^
		14	88.14 ± 5.46^a^	112.24 ± 6.95^a-c^	15.65 ± 0.97^a^	95.01 ± 5.88^a^	38.33 ± 2.38^a^	120.87 ± 7.48^a^	169.56 ± 10.49^a^
		28	87.94 ± 5.44^a^	112.08 ± 6.94^a-c^	15.58 ± 0.97^a^	93.24 ± 5.77^a^	38.00 ± 2.36^a^	120.24 ± 7.44^a^	166.89 ± 10.33^a^
	N_1_	0	91.12 ± 5.64^a^	116.14 ± 7.19^a^	14.18 ± 0.88^b^	94.51 ± 5.85^a^	39.21 ± 2.43^a^	122.02 ± 7.55^a^	172.87 ± 10.70^a^
		14	84.56 ± 5.23^a^	108.14 ± 6.69^bc^	15.44 ± 0.96^a^	87.95 ± 5.44^b^	39.33 ± 2.44^a^	118.31 ± 7.32^a^	154.35 ± 09.55^b^
		28	82.19 ± 5.09^a^	106.47 ± 6.59^c^	16.14 ± 1.00^a^	82.88 ± 5.13^b^	39.91 ± 2.47^a^	116.75 ± 7.23^a^	146.58 ± 09.07^b^
Diabetic subjects (Study 2)	D_0_	0	131.24 ± 4.90^ab^	215.24 ± 8.03^ab^	14.26 ± 0.54^c^	125.31 ± 4.68^a^	34.03 ± 1.27^bc^	145.12 ± 5.41^a^	223.15 ± 08.32^a^
		14	127.11 ± 4.74^bc^	209.08 ± 7.80^bc^	14.78 ± 0.56^b^	123.15 ± 4.59^a^	33.12 ± 1.24 ^cd^	144.74 ± 5.40^a^	215.87 ± 08.05^b^
		28	125.82 ± 4.69^c^	206.92 ± 7.72 ^cd^	15.09 ± 0.57^b^	122.87 ± 4.58^a^	32.17 ± 1.20^d^	143.12 ± 5.34^a^	206.84 ± 07.71^c^
	D_1_	0	132.17 ± 4.93^a^	218.24 ± 8.14^a^	14.70 ± 0.55^bc^	124.14 ± 4.63^a^	33.21 ± 1.24^b-d^	146.01 ± 5.45^a^	224.32 ± 08.36^a^
		14	125.15 ± 4.67^c^	201.11 ± 7.50^d^	16.25 ± 0.61^a^	117.01 ± 4.37^b^	34.29 ± 1.28^b^	141.29 ± 5.27^a^	207.21 ± 07.73^c^
		28	117.04 ± 4.37^d^	176.45 ± 6.58^e^	16.51 ± 0.62^a^	105.09 ± 3.92^c^	36.15 ± 1.35^a^	139.71 ± 5.21^a^	195.99 ± 07.31^d^

Values with similar alphabetical letters in a column are statistically alike (P > 0.05) only within respective study.

N_0_ = Normal Control; N_1_ = Normal subjects consuming designer biscuit; D_0_ = Diabetic Control; D_1_ = Diabetic subjects consuming designer biscuit.

##### 3.5.1.2 Random serum glucose

Treatment, intervals, and their interactions were statistically significant in study 2, while no significance with respect to treatment and interaction was observed in Study 1 although the intervals were significantly different. Effect of treatment on random serum glucose in D_0_ and D_1_ showed mean value of 210.42 ± 8.38 and 198.6 ± 18.87 mg/dL respectively (Table 5). Pooled means for the effect of intervals on random serum glucose of normal and diabetic individuals are mentioned in Table 6. At day 0, the maximum value of random serum glucose by normal and diabetic individuals was recorded as 115.13 ± 7.01 mg/dL and 216.74 ± 8.02 mg/dL, respectively, while minimum values of random serum glucose in D_0_ and D_1_ recorded at day 28 were 109.28 ± 7.19 mg/dL and 191.68 ± 7.12 mg/dL, respectively. With the designer biscuits provision, random serum glucose level of diabetic subjects reduced from 218.24 ± 8.14 mg/dL to 176.45 ± 6.58 mg/dL, whereas it dropped from 215.24 ± 8.03 mg/dL to 206.92 ± 7.72 mg/dL after 28 days without the provision of designer biscuits (Table 7). The reduction percentage of random glucose with the consumption of designer biscuits in normal subjects was 8.326 %, while that of diabetic subjects was 19.14 %.

##### 3.5.1.3 Serum insulin

Statistical analysis regarding insulin explained that the designer biscuits momentously affected insulin levels. In Study 2, the treatments as well as intervals were highly significant (P < 0.01) with respect to insulin levels while in Study 1, the treatments were non-significant (P > 0.05), but intervals showed significant effect (P < 0.05). Mean value for effect of treatment on insulin in D_0_ was observed as 14.71 ± 0.64 IU/L, while that in D_1_ was 15.82 ± 1.00 IU/L (Table 5). Pooled mean for intervals effect on insulin levels of normal subjects at 0 day was 15.05 ± 1.27 IU/L, which increased to 15.86 ± 1.00 IU/L after 28 days, while diabetic subjects at initial day showed insulin level of 14.48 ± 0.58 IU/L which increased to 15.80 ± 0.93 IU/L at final day (Table 6). From the interactive means of treatment and intervals as shown in Table 7, it can be observed that in study 1, the normal control subjects showed decrease in insulin level from 15.91 ± 0.99 IU/L to 15.58 ± 0.97 IU/L, whereas an increasing trend was observed over 28 days with the designer biscuit provision from 14.18 ± 0.88 IU/L to 16.14 ± 1.00 IU/L. D_0_ represented increase in insulin levels over 28 days from 14.26 ± 0.54 to 15.09 ± 0.57 IU/L while the increase in D_1_ after treatment provision for 2 weeks period was observed as 14.70 ± 0.55 IU/L at day 0 and 16.51 ± 0.62 IU/L at day 28. The percentage increase in insulin level of diabetic subjects in study 2 was 12.31 % after they consumed the treatment fortnightly.

#### 3.5.2 Lipid profile

##### 3.5.2.1 Serum low density lipoproteins (LDL)

The treatment, time intervals and interaction of both studies were significantly different. The pooled means for treatment effect represents that control and treated group of study 1 had LDL values 94.47 ± 5.71 and 88.45 ± 7.17 mg/dL, respectively, while LDL values for control and treated group of study 2 were 123.78 ± 4.59 and 115.42 ± 9.02 mg/dL, respectively (Table 5). Mean values for the effect of intervals on LDL as shown in Table 6 represents that in study 1, normal subjects at 0 day had mean LDL levels of 94.83 ± 5.72 mg/dL, while at day 28 the mean LDL level was observed as 88.06 ± 7.52 mg/dL. In study 2, diabetic subjects showed mean serum LDL levels at day 0 as 124.73 ± 4.57 mg/dL, while at day 28 the observed mean LDL level was 113.98 ± 10.03 mg/dL. Means values for interaction of treatment and intervals as summarized in Table 7 describe that normal subjects at beginning of study had mean LDL values as 95.14 ± 5.89 mg/dL, which dropped to 93.24 ± 5.77 mg/dL at the end of study. The normal subjects consuming designer biscuits showed decreased LDL levels over 28 days from 94.51 ± 5.85 to 82.88 ± 5.13 mg/dL. Their percentage reduction was observed as 12.3 %. Mean LDL levels for control diabetic subjects at day 0 were 125.31 ± 4.68 mg/dL that dropped to 122.87 ± 4.58 mg/dL after 28 days. Diabetic subjects with designer biscuits intervention showed mean LDL levels dropped in 28 days from 124.14 ± 4.63 mg/dL to 105.09 ± 3.92 mg/dL. The percentage reduction of 15.35 % occurred within twenty-eight days of study in diabetic subjects.

##### 3.5.2.2 Serum high density lipoproteins (HDL)

Results pertaining to treatment and interaction of treatment and intervals for HDL levels were significant in study 2, while study 1 represented non-significant results with respect to treatment, time intervals and interaction of both. Mean value for the effect of treatment on HDL level of control diabetic individuals was observed as 33.11 ± 1.42 mg/dL whereas that of treated diabetic individuals was 34.55 ± 1.75 (Table 5). Mean HDL values of control diabetic individuals at day 0 and day 28 were 34.03 ± 1.27 mg/dL and 32.17 ± 1.20 mg/dL, respectively (Table 6). While the HDL levels of diabetic individuals consuming the treatment at day 0 were 33.21 ± 1.24 mg/dL that rose to 36.15 ± 1.35 mg/dL after 28 days (Table 7). Effect of treatment over a period of 28 days represents the percentage difference that normal group of subjects who had the designer biscuits treatment showed 2.22 % increase in the serum HDL levels, while the treated diabetic individuals showed 8.85 % increase in the serum levels of high-density lipoproteins.

##### 3.5.2.3 Serum triglyceride

The results showed that treatment, time intervals and interaction of treatment and time intervals were not significant in either of the studies. As shown in Table 7, mean values of serum triglyceride levels in normal subjects at initiation of treatment were 121.79 ± 7.34 mg/dL that dropped to 118.50 ± 7.36 mg/dL at the end of the trial. In diabetic subjects, pooled mean value of serum triglyceride was observed as 145.57 ± 5.30 mg/dL in the beginning that dropped to 141.42 ± 5.43 mg/dL when the trial ended.

##### 3.5.2.4 Total cholesterol

Treatments and intervals showed significant impact on total cholesterol exhibiting a decreasing trend in both studies. Interaction of treatments and intervals also produced significant results in both normal and diabetic individuals. The effect of treatment on total cholesterol level as expressed in terms of pooled means of N_0_ showed 169.73 ± 10.42 mg/dL while N_1_ was 157.94 ± 14.67 mg/dL. Mean value for effect of treatment on total cholesterol of diabetic control subjects was observed as 215.29 ± 10.3 mg/dL and 209.18 ± 14.05 mg/dL was observed in diabetic subjects consuming designer biscuits (Table 5). The maximum value (172.81 ± 10.41) mg/dL of total cholesterol in normal subjects was recorded at day 0, while minimum value (156.74 ± 14.07 mg/dL) was observed after 28 days (Table 6). Diabetic subjects showed elevated levels of serum cholesterol 223.74 ± 8.14 mg/dL at day 0, which reduced in 28 days to 201.42 ± 9.19 mg/dL. With the provision of designer biscuits on fortnightly basis, total cholesterol of normal and diabetic individuals reduced from 172.87 ± 10.70 mg/dL to 146.58 ± 9.07 mg/dL and 224.32 ± 8.36 mg/dL to 195.99 ± 7.31 mg/dL, respectively after 28 days (Table 7).

#### 3.5.3 Hepatic health profile

##### 3.5.3.1 Aspartate aminotransferase (AST)

The treatments and interval in both studies were statistically significant and exhibited a decreasing trend in AST level. Interaction of treatment and intervals were statistically significant in normal study, while it was non-significant in diabetic study. The mean value of AST as an effect of treatment in N_0_ was recorded as 31.00 ± 1.91 mg/dL, while in N_1_ it was 32.99 ± 3.00 mg/dL. D_0_ and D_1_ showed mean AST values for effect of treatment as 38.18 ± 1.84 mg/dL and 35.44 ± 2.37 mg/dL, respectively (Table 8). The effect of intervals on AST levels of normal and diabetic subjects is shown in Table 9. Mean value of 33.61 ± 2.94 mg/dL was recorded at day 0 that reduced to 30.36 ± 1.84 mg/dL at final day in normal subjects whereas in diabetic subjects, mean value of 38.82 ± 1.69 mg/dL was recorded at day 0 and 34.98 ± 2.29 mg/dL was observed at day 28. According to the mean values mentioned in Table 10, in the beginning of the treatment, AST level of the normal subjects without the intervention of designer biscuits was 31.54 ± 1.96 mg/dL that decreased to 30.48 ± 1.89 mg/dL at the end of the treatment. AST levels of normal subjects that consumed designer biscuits dropped from 35.67 ± 2.21 mg/dL to 30.25 ± 1.88 mg/dL after 28 days. In Study 2, at initiation, the maximum recorded mean AST value of diabetic individuals not consuming the treatment was observed as 39.73 ± 1.49 mg/dL and minimum value observed at day 28 was 36.83 ± 1.38 mg/dL whereas the diabetic individuals consuming designer biscuits had mean value of AST as 37.91 ± 1.42 mg/dL at day 0 that dropped to 33.13 ± 1.24 mg/dL at day 28. In normal study, 15.2 % reduction occurred in AST levels because of the treatment. While study 2 showed a reduction of 12.61 % with the provision of designer biscuits on fortnightly basis.

**Table 8:** Pooled means for effect of treatments on hepatic and renal health indicators of normal and diabetic individuals treated with pulses-supplemented designer biscuits.

**Studies**	**Treatments**	**Aspartate aminotransferase** **(mg/dL)**	**Alanine aminotransferase** **(mg/dL)**	**Alkaline phosphatase** **(mg/dL)**	**Bilirubin** **(mg/dL)**	**Urea** **(mg/dL)**	**Creatinine** **(mg/dL)**
Normal subjects (Study I)	N_0_	31.00 ± 1.91^b^	29.37 ± 2.06^b^	164.06 ± 10.13^a^	0.84 ± 0.06^a^	42.58 ± 3.04^a^	0.80 ± 0.05^a^
	N_1_	32.99 ± 3.00^a^	31.86 ± 2.37^a^	164.63 ± 10.04^a^	0.83 ± 0.05^a^	39.09 ± 2.39^b^	0.79 ± 0.05^a^
Diabetic subjects (Study II)	D_0_	38.18 ± 1.84^a^	38.93 ± 1.9^a^	172.43 ± 6.48^a^	0.94 ± 0.04^a^	49.59 ± 2.20^a^	1.09 ± 0.06^a^
	D_1_	35.44 ± 2.37^b^	37.59 ± 2.54^b^	172.23 ± 6.84^a^	0.87 ± 0.07^b^	45.50 ± 2.38^b^	1.04 ± 0.07^b^

Values with similar alphabetical letters in a column are statistically alike (P > 0.05) only within respective study.

N_0_ = Normal Control; N_1_ = Normal subjects consuming designer biscuit; D_0_ = Diabetic Control; D_1_ = Diabetic subjects consuming designer biscuit.

**Table 9:** Pooled means for effect of intervals on hepatic and renal health indicators of normal and diabetic individuals treated with pulses-supplemented designer biscuits.

**Studies**	**Intervals** **(days)**	**Aspartate aminotransferase** **(mg/dL)**	**Alanine aminotransferase** **(mg/dL)**	**Alkaline phosphatase** **(mg/dL)**	**Bilirubin** **(mg/dL)**	**Urea** **(mg/dL)**	**Creatinine** **(mg/dL)**
Normal subjects (Study I)	0	33.61 ± 2.94^a^	32.09 ± 2.42^a^	166.80 ± 10.05^a^	0.83 ± 0.05^a^	40.00 ± 2.40^a^	0.80 ± 0.05^a^
	14	32.01 ± 2.20^b^	30.03 ± 2.70^b^	162.54 ± 10.00^a^	0.83 ± 0.06^a^	41.29 ± 3.30^a^	0.79 ± 0.05^a^
	28	30.36 ± 1.84^c^	29.71 ± 1.84^b^	163.69 ± 09.97^a^	0.83 ± 0.06^a^	41.21 ± 3.77^a^	0.79 ± 0.05^a^
Diabetic subjects (Study II)	0	38.82 ± 1.69^a^	38.39 ± 1.65^ab^	173.54 ± 06.84^a^	0.94 ± 0.04^a^	47.78 ± 1.84^a^	1.08 ± 0.06^a^
	14	36.63 ± 1.92^b^	38.80 ± 1.42^a^	171.18 ± 06.22^a^	0.90 ± 0.06^b^	47.59 ± 2.30^a^	1.06 ± 0.06^a^
	28	34.98 ± 2.29^c^	37.60 ± 3.36^b^	172.25 ± 06.86^a^	0.88 ± 0.09^c^	47.25 ± 4.53^a^	1.05 ± 0.09^a^

Values with similar alphabetical letters in a column are statistically alike (P > 0.05) only within respective study.

**Table 10:** Hepatic and renal health indicators of normal and diabetic individuals treated with pulses-supplemented designer biscuits.

**Studies**	**Groups**	**Intervals** **(days)**	**Aspartate aminotransferase** **(mg/dL)**	**Alanine aminotransferase** **(mg/dL)**	**Alkaline phosphatase** **(mg/dL)**	**Bilirubin** **(mg/dL)**	**Urea** **(mg/dL)**	**Creatinine** **(mg/dL)**
Normal subjects (Study 1)	N_0_	0	31.54 ± 1.96^bc^	30.68 ± 1.90^bc^	166.47 ± 10.30^a^	0.82 ± 0.06^a^	40.29 ± 2.50^b^	0.79 ± 0.05^a^
		14	30.98 ± 1.92^c^	28.09 ± 1.74^d^	160.57 ± 09.94^a^	0.84 ± 0.06^a^	43.47 ± 2.69^a^	0.79 ± 0.05^a^
		28	30.48 ± 1.89^c^	29.32 ± 1.82 ^cd^	165.13 ± 10.22^a^	0.84 ± 0.06^a^	43.96 ± 2.72^a^	0.81 ± 0.05^a^
	N_1_	0	35.67 ± 2.21^a^	33.49 ± 2.08^a^	167.12 ± 10.34^a^	0.83 ± 0.06^a^	39.70 ± 2.46^a^	0.80 ± 0.05^a^
		14	33.04 ± 2.05^b^	31.97 ± 1.98^ab^	164.51 ± 10.18^a^	0.82 ± 0.06^a^	39.11 ± 2.42^a^	0.79 ± 0.05^a^
		28	30.25 ± 1.88^c^	30.10 ± 1.87^c^	162.26 ± 10.04^a^	0.82 ± 0.06^a^	38.46 ± 2.38^a^	0.78 ± 0.05^a^
Diabetic subjects (Study 2)	D_0_	0	39.73 ± 1.49^a^	37.53 ± 1.40^c^	170.94 ± 06.38^a^	0.93 ± 0.04^a^	48.37 ± 1.81^bc^	1.04 ± 0.04^b^
		14	37.97 ± 1.42^a^	38.68 ± 1.45^bc^	171.33 ± 06.39^a^	0.94 ± 0.04^a^	49.06 ± 1.83^b^	1.09 ± 0.05^a^
		28	36.83 ± 1.38^a^	40.58 ± 1.52^a^	175.00 ± 06.53^a^	0.95 ± 0.04^a^	51.33 ± 1.92^a^	1.13 ± 0.05^a^
	D_1_	0	37.91 ± 1.42^a^	39.24 ± 1.47^b^	176.14 ± 06.57^a^	0.94 ± 0.04^a^	47.19 ± 1.76 ^cd^	1.11 ± 0.05^a^
		14	35.28 ± 1.32^a^	38.91 ± 1.46^b^	171.03 ± 06.38^ab^	0.86 ± 0.04^b^	46.12 ± 1.72^d^	1.02 ± 0.04^b^
		28	33.13 ± 1.24^a^	34.61 ± 1.29^a^	169.50 ± 06.32^b^	0.81 ± 0.03^c^	43.18 ± 1.61^e^	0.98 ± 0.04^c^

Values with similar alphabetical letters in a column are statistically alike (P > 0.05) only within respective study.

N_0_ = Normal Control; N_1_ = Normal subjects consuming designer biscuit; D_0_ = Diabetic Control; D_1_ = Diabetic subjects consuming designer biscuit.

##### 3.5.3.2 Alanine aminotransferase (ALT)

Treatment showed significant impact on ALT level exhibiting a decreasing trend in both normal and diabetic subjects. Intervals also exhibited significant results in both studies. Interaction of treatment and time intervals also produced significant results in normal as well as diabetic individuals. Effect of treatment on ALT in N0 and N1 showed mean value of 29.37 ± 2.06 and 31.86 ± 2.37 mg/dL, respectively, while D_0_ and D_1_ showed mean value of 38.93 ± 1.9 and 37.59 ± 2.54 mg/dL, respectively (Table 8). Mean values of ALT levels summarized in Table 9 indicate the effect of intervals on normal subjects as 32.09 ± 2.42 mg/dL at day 0 that dropped to 29.71 ± 1.84 mg/dL at day 28, while that of diabetic subjects were observed as 38.82 ± 1.69 mg/dL at day0 and 34.98 ± 2.29 mg/dL at the day 28. During the study, at Day 0 the ALT level of control subjects decreased from 30.68 ± 1.90 mg/dL to 29.32 ± 1.82 mg/dL after 28 days and that of diabetic subjects decreased from 37.53 ± 1.40 to 40.58 ± 1.52 mg/dL. With designer biscuit incorporation, there was a reduction from 33.49 ± 2.08 to 30.10 ± 1.87 mg/dL over the 28 days period in normal subjects, while a reduction from 37.91 ± 1.42 to 33.13 ± 1.24 mg/dL over 28 days was observed in diabetic subjects (Table 10). The percentage decrease of 10.12 % in normal individuals who had the treatment, while that of 11.8 % was observed in diabetic individuals, who consumed designer biscuits.

##### 3.5.3.3 Alkaline phosphatase (ALP)

The statistical analysis revealed that both the treatment and intervals were non-significant in both studies. Also, the interaction of treatment and intervals were non-significant in normal study, while interaction was significant in diabetic study. Diabetic subjects with designer biscuits provision showed mean ALP level of 176.14 ± 06.57 mg/dL in the beginning of treatment and 169.50 ± 06.32 mg/dL as the treatment ended. The percentage decrease observed in normal and diabetic subjects who had the treatment was 2.9 % and 3.7 %, respectively.

##### 3.5.3.4 Bilirubin

Both treatment and time intervals in study 2 were statistically significant, while no significant difference was observed in study 1 with respect to treatment and time intervals. Interaction was also significant in diabetic subjects but non-significant in normal subjects. Table 8 summarizes the pooled mean values for the effect of treatment on bilirubin levels. The values observed in D_0_ and D_1_ were 0.94 ± 0.04 and 0.87 ± 0.07 mg/dL, respectively. In study 2, at day 0 the mean values for bilirubin levels of diabetic subjects were 0.94 ± 0.04 mg/dL that reduced to 0.88 ± 0.09 mg/dL after 28 days. Diabetic subjects consuming designer biscuits represented mean bilirubin value at day 0 as 0.94 ± 0.04 mg/dL that lowered over 28 days to 0.81 ± 0.03 mg/dL. Diabetic patients who received the treatment showed a 13.83 % decrease in their serum bilirubin level after 28 days.

#### 3.5.4 Renal health profile

##### 3.5.4.1 Urea

Statistical analysis of data showed a significant difference of the treatments in both normal and diabetic subjects, while the intervals were not significant in both studies. Treatment and intervals also interactively produced significant results in normal as well as diabetic study. Table 8 indicates mean values for the effect of treatment on urea levels of normal and diabetic subjects. Mean value for urea level in N_0_ and N_1_ was 42.58 ± 3.04 mg/dL and 39.09 ± 2.39 mg/dL, respectively. Treatment effect on urea level of D_0_ depicted mean value 49.59 ± 2.20 mg/dL and that of D_1_ was 45.50 ± 2.38 mg/dL. In diabetic control study, minor increase was observed because at day 0 the mean urea level was 48.37 ± 1.81 mg/dL and 51.33 ± 1.92 mg/dL at day 28. Diabetic study with intervention of designer biscuits represented a decrease in urea level with mean value at treatment initiation 47.19 ± 1.76 mg/dL that reduced to 43.18 ± 1.61 mg/dL at end of the study. The percentage change in urea levels of both normal and diabetic individuals. In study 1, the percentage reduction in urea level of individuals consuming designer biscuits was 3.12 %, while diabetic subjects in study 2 consuming designer biscuits showed a percentage decrease of 8.49 % after 28 days.

##### 3.5.4.2 Creatinine

Statistical analysis showed that treatment and interaction were significantly different in diabetic study, while intervals were non-significant. In normal study, treatments, intervals, and interaction were non-significant. Table 8 summarizes pooled means for the effect of treatment on creatinine level of diabetic subjects. Mean value of creatinine level in D_0_ and D_1_ was observed as 1.09 ± 0.06 mg/dL and 1.04 ± 0.07 mg/dL, respectively. In control diabetic subjects, interaction of treatment and intervals produced significant impact on creatinine levels exhibiting an increasing trend. At 0-day, the mean creatinine level of diabetic control subjects was observed as 1.04 ± 0.04 mg/dL and at day 28 it was 1.13 ± 0.05 mg/dL. On the other hand, diabetic subjects consuming designer biscuits showed a mean creatinine value of 1.11 ± 0.05 mg/dL at day 0 that dropped after 28 days and showed a mean value 0.98 ± 0.04 mg/dL (Table 10). Percentage reduction in normal subjects with treatment provision was 2.5 %, while 11.7 % reduction was observed in diabetic subjects consuming designer biscuits.

## 4 Discussion

### 4.1 Compositional profile

The study showed that designer biscuits composed of wheat and pulse-supplemented mixtures were highly acceptable when compared with control and chosen for proximate analysis. It can be deduced, on the basis of proximate analysis of prepared designer biscuits, that pulse flour incorporation increased the nutritional status of biscuits because of high protein content. Aleem Zaker, Genitha, and Hashmi (2012) prepared biscuits with different concentrations of defatted soy flour and the analysis of its proximate composition revealed that increased concentration of defatted soy flour significantly effects the proximate composition. Another study by Gbenga‐Fabusiwa, Oladele, Oboh, Adefegha, and Oshodi (2018) produced similar results with that of current study regarding proximate analysis, their biscuits were produced from pigeon pea-wheat based composite flour.

Rich mineral content in pulses may be attributed to this marginally higher ash content (Gbenga‐Fabusiwa, Oladele, Oboh, Adefegha, and Oshodi, 2018). Fat content of designer biscuits containing 50 % chickpea was highest compared to rest of prepared treatments, whereas biscuits containing mung bean flour showed variable trend compared to control. Leavening effect might be the main reason for these observed differences in fat contents (Ismail, Sahri, & Abd Hamid, 2018). Varying proportion of ingredients in designer biscuits caused increased variation in fiber content providing/promising several healthy benefits like colonic digestion and reduction in constipation which is otherwise associated with regular biscuits (Makinde & Taibat, 2018). Incorporation of pulse flour in wheat flour decreased the total carbohydrate content, as they constitute complex carbohydrates, rich proteins, and fibers. Similar results were reported by (Soni, Kulkarni, & Patel, 2018).

As compared to control sample, low moisture content was observed in other treatments of prepared designer biscuits. Soni, Kulkarni, and Patel (2018) also observed a similar trend in moisture content of prepared designer biscuit lowered with increase in supplementation of pulse flour. The reason is the fact that pulse flour absorbs moisture in baked products. Higher levels of moisture content indicate shorter shelf life of designer biscuits made of composite flour as they encourage microbial growth leading to spoilage (N. Mishra and Chandra, 2012, Mishra et al., 2012).

Ash content indicates an estimation of total mineral content in a given quantity of food substance. Positive correlation was found in ash content with the supplementation of pulse flour into wheat flour. Gbenga‐Fabusiwa, Oladele, Oboh, Adefegha, and Oshodi (2018) also observed the same correlation, their prepared biscuits had higher ash content as pigeon pea flour was incorporated to wheat flour. In this study, pulses i.e.*,* chickpea and mung bean flours were added to wheat flour. Another reason for improvement in ash content level is due to presence of minerals within pulses.

The protein content of designer biscuits increased with the increase in incorporation of pulse based composite flour. Pulses are an excellent source of protein and complement to cereal protein that are limited in lysine. Pulse flour addition not only improves quality but also the quantity of protein content within food products thereby has great potential in combating with health concerns like protein energy malnutrition. Banureka and Mahendran (2009) and N. Mishra and Chandra, 2012, Mishra et al., 2012 reported similar trends where protein content of most preferred designer biscuits in this study were higher as compared to the control. High content of proteins and fibers in pulse flour contributes greatly to water holding capacity in composite flours (Jeong & Chung, 2019) due to which there was slight decrease observed with moisture content in prepared designer biscuits.

A slight decrease was observed in fat content. Previous report has stated that chickpea has lower fat content therefore, replacement of chickpea flour in product decreases the fat content (Aleem Zaker, Genitha, & Hashmi, 2012). For the creaming process in making good biscuits, plasticity of fats is crucial as it entraps air and retains its ampule volume that contributes to the leavening effect. Solid fat content produces low consistency dough that easily expanded during baking and gas release (Ismail, Sahri, & Abd Hamid, 2018). Results of this study follow the results reported by their study.

Due to complex carbohydrates and high fibers content of pulses, it was reasonable to use their flour for manufacturing biscuits so that health-promoting benefits of their nutrients can be enriched into prepared designer biscuits (Krystyjan, Gumul, Korus, Korus, & Sikora, 2018). Total carbohydrate content decreased with the increased level of chickpea and mungbean based pulse flour. This may be attributed due to the replacement of wheat flour with pulse flour as pulses are rich in protein. These results are similar with the results reported by Soni, Kulkarni, and Patel (2018). Absorption and digestion of dietary carbohydrates take place in small intestines. Simple sugars like glucose and fructose are absorbed directly in small intestine whereas the complex sugars as present in wheat or pulse flour have glycosidic bond in them that needs to be hydrolyzed first so that it releases its monosaccharaides because they are too large to be absorbed directly (Kendall, 2018).

### 4.2 Physical parameters

It was discovered through the results that increase in level of incorporation of pulse-based flour caused increase of total weight, diameter and spread ratio. Various researchers including Soni et al., 2018, Waleed et al., 2018 and Ismail, Sahri, and Abd Hamid (2018) analyzed physical characteristics of biscuits made of different formulations that produced similar results to our study**.** Higher weight of designer biscuits may be due to the oil retaining ability of wheat flour during baking process as described by Waleed, Mahdi, Li, Qian, and Wang (2018). Slight difference was observed in diameter and height of three best-selected designer biscuits treatments however they height showed a statistically significant difference whereas diameter was insignificant.

One of the important parameters in quality determination of biscuits is the diameter or spread ability. This may be related to the fat content in biscuits. As biscuits contained solid shortening, therefore, the diameter increased. Similar trend was reported by Ismail, Sahri, and Abd Hamid (2018) when they analyzed the effect of shortening on physical properties of biscuits. Fats added in preparation of biscuits act as shortening agents that can double the biscuits in terms of height. As compared to the control treatment, height in other treatments of designer biscuits decreased. This reducing trend of height disagrees with Ismail, Sahri, and Abd Hamid (2018) but completely agrees with the results reported by Waleed, Mahdi, Li, Qian, and Wang (2018). The increase in spread factor may be attributed to better binding strength of pulses protein that also resulted in increasing height of designer biscuits. Soni, Kulkarni, and Patel (2018) reported that replacement levels of 0–25 % in wheat flour were observed to cause significant change in spread factor. The results obtained also agreed with this reported result. The viscosity of dough subsequently decreases with the substitution of non-wheat flour as gluten protein lowers. Consequently, less viscous dough results in high spread factor contributing to larger diameter of composite flour biscuits (Makinde & Taibat, 2018).

### 4.3 Sensory evaluation

In this research, the mean score of color declined because of increased substitution level. Soni, Kulkarni, and Patel (2018) also reported better color scores of cookies incorporated with chickpea followed by control cookies. As substitution was increased, the color of biscuits turned from light brown to dark brown that lead to lower acceptance. The darker color may be because of Maillard’s reaction that is a non-enzymatic reaction between lysine protein and reducing sugar molecules. Mung bean and chickpea are known to be rich in lysine that produces darker shades of brown color. Several authors reported darker colors of breads and biscuits that are fortified (N. Mishra and Chandra, 2012, Mishra et al., 2012, Soni et al., 2018, Waleed et al., 2018). Similarly, mean score of taste decreased with increased substitution level. The designer biscuit that was scored better among other treatments was prepared by the incorporation of 12.5 % chickpea and mung bean-based flours in 75 % wheat flour. Personal preference may be one reason, but the main reason can be the fact that consumption of chickpea flour in many forms is a common practice. Therefore, the consumers subjectively ranked it better as compared to other treatments. Waleed et al. (2018) also reported similar trend for subjective scores of panelists. Relative decrease in the mean scores for aroma may be due to the pulse flour incorporation because they do not have a very acceptable aroma. Also, the quality of raw materials used in biscuits processing influences the aroma characteristic. The presence of vanilla essence significantly influenced the aroma characteristic of prepared designer biscuits. Alam, Alam, Hakim, Huq, and Moktadir (2014) reported that biscuits’ texture depends mainly on sugar proportion used and rate of dough development. Textural characteristics of designer biscuits change significantly by variation in the inclusion ratio of different pulses over the control sample. Results obtained agree with the results reported by Soni, Kulkarni, and Patel (2018) and Waleed, Mahdi, Li, Qian, and Wang (2018) since their product secured similar mean scores in terms of texture as the current study. The highest mean scores of mouth feel were secured by those treatments of designer biscuits that contained 25 % integration of pulse flours. Designer biscuits securing high as compared to others were observed to be better in hardness as mentioned by Alam, Alam, Hakim, Huq, and Moktadir (2014). Overall acceptability of designer biscuits was justified with the textural profile. Up to 25 % incorporation of composite flour to designer biscuits showed slight improvement in crispiness of biscuits hence better scores were secured as compared to the designer biscuits containing 50 % of incorporated composite flour. Dryness of mouth was reported by the panelists hence minimum scores were secured by such biscuits. On the basis of designer biscuit’s overall acceptability, this can be suggested that incorporation of 12.5 % of chickpea and mungbean flours in composite flour preparation is superior to control sample and in all other treatments with respect to sensorial quality characteristics such incorporation could be considered optimum. Soni, Kulkarni, and Patel (2018) also observed the same pattern for overall acceptability as observed in our study and their results somehow agrees as well.

### 4.4 Glycemic and satiety indices

Mean GI of treatments of designer biscuits was extremely lower as compared to the control. As reported by Gbenga‐Fabusiwa, Oladele, Oboh, Adefegha, and Oshodi (2018) biscuits prepared with substitution of pigeon pea flour represented low glycemic index. The reason for lowering of glycemic index in designer biscuits can be attributed to the addition of pulses that contain 5–10 % more amylose compared to cereal grains. This amylose is more resistant to digestion. Incorporation of pulses increases protein content, and these high amounts of proteins encapsulate starch that prevents the access of enzyme. Apart from the amylose and protein contents, crude fiber content also increased in all enriched designer biscuits. Dietary fibers also have the ability to inhibit digestibility of starch by increasing intestinal contents’ viscosity eventually slowing carbohydrate absorption from food (Banureka & Mahendran, 2009). It has been observed that dietary carbohydrates influence satiety, however, there is no clarity regarding carbohydrate sources as primary determinants of satiety. Present study results suggest that based on sensory quality attributes, best selected treatments had raised satiety indices therefore it can be concluded that treatment source is the satiety determinant. These results completely agree with the results represented by Zhuoshi Zhang, Venn, Monro, and Mishra (2018). Insulin is also an important element of complex mechanisms influencing energy intake. It not only acts to regulate long-term food intake but short-term satiety as well. Low insulin levels sensed by the brain elicit response of hunger and eventually leads to search of an energy source (Kendall, 2018).

### 4.5 Glycemic and serum lipid profile of subjects

Diabetes induction leads to an increase in blood glucose levels. However, feeding with designer biscuits led to a significant decrease in both fasting and random blood glucose levels depicting hypoglycemic activity in treated diabetic individuals. These results conformed to a similar study conducted on rats (Erukainure, Ebuehi, Adeboyejo, Okafor, Muhammad, & Elemo, 2013). According to a recent research by Irondi, Ajani, Aliyu, Olatoye, Abdulameed, and Ogbebor (2021), yellow maize (*Zea mays* L.) and cowpea (*Vigna unguiculata* L. Walp) composite biscuits have shown enzyme (α-amylase and α-glucosidase) inhibitory activity that may deliver lower glycemic response of the product. The scientists suggested that these composite biscuits may be considered as potential functional food to address obesity and type-2 diabetes (Irondi, Ajani, Aliyu, Olatoye, Abdulameed, & Ogbebor, 2021). However, in current research, the enzyme inhibitory activities of the designer biscuits were not studied but their glycemic and satiety profiles were investigated. Since, maintenance of normal blood glucose levels must be the goal for diabetic individuals so it is necessary to control the intake of high glycemic foods. Results of current study favor this maintenance goal in compliance with the outcomes described by Sharma, Sharma, Singh, Kumar, and Tripathi (2018). Increased serum insulin levels indicated insulin release from pancreatic beta-cells responsible for hypoglycemic activity as inverse association and positive regression between serum glucose is evident (Erukainure, Ebuehi, Adeboyejo, Okafor, Muhammad, & Elemo, 2013). Non-insulin dependent diabetes mellitus (NIDDM) often occurs because of beta cell function failure in the presence of chronic insulin resistance. This correlates with current observation that in normal study, untreated subjects showed decreased insulin level, while the treated individuals consuming the designer biscuits had increased beta-cell function that correlates with observed increase in the insulin level. However, a pulse-based diet study by Kazemi, McBreairty, Chizen, Pierson, Chilibeck, and Zello (2018) resulted in decreasing insulin, which disagrees with results of current study. The possible causes could be differences in the study participants, design, and intervention. However, further studies can be made to confirm the effect, keeping in view other co-factors.

Insulin resistance also influences number of changes in lipid metabolism. In diabetic patients, leading cause of cardiovascular diseases are dyslipidemia and is common among NIDDM patients. Some studies have reported that fortified biscuits may also improve lipid profile in patients with diabetes mellitus (Ejtahed, Mohtadi-Nia, Homayouni-Rad, Niafar, Asghari-Jafarabadi, Mofid, et al., 2011; Erukainure, Ebuehi, Adeboyejo, Okafor, Muhammad, & Elemo, 2013). Designed present study investigated effects of biscuits consumption on lipid profile. It was shown that designer biscuits consumption improved the lipid profile in terms of lowering LDL levels and total cholesterol and increasing the serum HDL levels compared with the normal biscuits. Serum triglycerides level remained unchanged in the intervention group during the study that resembles with the results of Ejtahed, et al. (2011). Results of another pulse-based diet study were in accordance with current study, which concluded that a low glycemic index diet effectively decreases LDL and increases HDL levels. LDL lowering effect of pulse-based diet was described as more efficient. Reduction in total cholesterol was also in agreement with the same study (Kazemi, McBreairty, Chizen, Pierson, Chilibeck, & Zello, 2018). In a study, Erukainure et al. (2015) observed parallel trend with current study that reduction in elevated levels of total cholesterol, triglycerides and LDL, as well as increased HDL occurred on consumption of formulated biscuits. This significance indicated antilipidemic activity.

### 4.6 Physiological compatibility

Diabetes is associated with liver dysfunction and high incidences of liver abnormalities are associated with diabetic individuals as compared to normal individuals. In regard to this statement, many studies worldwide have reported a varied occurrence of imbalances in liver function tests (LFT) among individuals with diabetes (Ghimire, Shakya, Shakya, Acharya, & Pardhe, 2018). This study aimed to figure out variation incidence among normal and diabetic individuals consuming designer biscuits. Decreasing trend of hepatic parameters was observed due to intervention through which it can be concluded that designer biscuits do not affect liver functioning rather maintains the hepatic health. High blood sugar level stresses kidneys to filter too much blood causing blood vessel damage leading to kidney disorder. Parameters for diagnosis of kidney functioning are urea and creatinine. Concentration of serum creatinine reliably reflects glomerulus filtration rate GFR as compared to concentration of serum urea. A rise in creatinine levels indicates kidney malfunctioning. Formation of urea is also influenced by many factors like intake of proteins, liver function and rate of protein catabolism (Sharma, Sharma, Singh, Kumar, & Tripathi, 2018). The hepatic and renal function tests were performed to check if there are any negative effects of the product’s consumption or not. It is noticed that the therapeutic regimen needs to be stopped as it is causing negative impacts on hepatic or renal health if the parameters are abnormally varying it. In current study, no abnormalities with respect to hepatic or renal functioning were seen. Conclusively, the results of the current study represented that pulses-supplemented designer biscuits did not negatively influence renal and hepatic health. Hence, these biscuits can be suggested for clinical trials at larger levels to ensure the effectiveness of such regime.

## 5 Conclusion

Based on the findings, it can be suggested that pulses-supplemented designer biscuits prepared for management of diabetes mellitus were effective as a functional food. The correlation analysis revealed that lower glycemic index of biscuits yields in higher satiety index. The designer biscuits prepared with 12.5 % incorporation of chickpea and mungbean pulses possessed hypoglycemic effect. The incorporation of pulses possessed hypoglycemic effects, resulting in a significant decrease in glucose level and increase in insulin level, which endorsed the modulatory effect of designer biscuits for diabetes mellitus. The altered biochemical profile in diabetic subjects was modulated with the consumption of developed biscuits, and no negative effects were recorded when diabetic subjects consumed these biscuits. The major limitation of the study could be the recruitment of subjects who were not taking any medication for the control of diabetes. Hence, large-scale study should be conducted to confirm the effects with more data and animal models can be helpful to discovering the underlying possible mechanisms of the noted effect alongside use of drugs in co-therapeutic.

## Funding

The open access publishing fee is covered under the agreement between the DEAL Consortium and Elsevier upon acceptance due to Shahida Anusha Siddiqui being affiliated to the Technical University of Munich.

## Institutional review board statement

The study was conducted according to the ethical principles of the Declaration of Helsinki, and all participants gave written informed consent. This clinical trial was registered with the Ethical Review Board, Imperial College of Business Studies, Lahore, Punjab, Pakistan (Registration No. PHND02163006, Dated 07-11-2018).

## Informed consent statement

The hypoglycemic effect of selected designer biscuit was assessed using experimental trials on diabetic human subjects after consent from participants and ethical approval from Ethical Review Board, Imperial College of Business Studies, Lahore, Punjab, Pakistan.

## Conflict of interest

The authors declare no conflict of interest.

## CRediT authorship contribution statement

**Iahtisham-Ul-Haq:** Writing – review & editing, Writing – original draft, Visualization, Software, Methodology, Investigation, Data curation. **Aqsa Akram:** Writing – original draft, Visualization, Software, Methodology, Investigation. **Iqra Yasmin:** Writing – review & editing, Writing – original draft, Supervision, Methodology, Investigation, Formal analysis, Conceptualization. **Hafiz Rizwan Sharif:** Writing – review & editing, Writing – original draft, Validation, Resources, Project administration, Formal analysis, Conceptualization. **Gulzar Ahmad Nayik:** Writing – review & editing, Validation, Supervision, Resources, Investigation, Data curation. **Seema Ramniwas:** Writing – review & editing, Methodology, Investigation, Formal analysis. **Shahida Anusha Siddiqui:** Writing – review & editing, Validation, Funding acquisition.

## Declaration of competing interest

The authors declare that they have no known competing financial interests or personal relationships that could have appeared to influence the work reported in this paper.

## Data Availability

Data will be made available on request. The data gathered in this study is included and discussed in the manuscript.
